# Secondary outcomes and qualitative findings of an open-label feasibility trial of lisdexamfetamine dimesylate for adults with bulimia nervosa

**DOI:** 10.1186/s40337-023-00796-x

**Published:** 2023-05-22

**Authors:** Laura Dixon, Sara Bartel, Victoria Brown, Sarrah I. Ali, Susan Gamberg, Andrea Murphy, Katherine L. Brewer, Susan L. McElroy, Allan Kaplan, Abraham Nunes, Aaron R. Keshen

**Affiliations:** 1 Eating Disorder Program, Nova Scotia Health Authority, Halifax, NS Canada; 2 Department of Psychiatry, Dalhousie University, Halifax, NS Canada; 3 College of Pharmacy, Dalhousie University, Halifax, NS Canada; 4 College of Doctoral Studies, University of Phoenix, Tempe, AZ USA; 5 Lindner Center of HOPE, Mason, OH USA; 6 Department of Psychiatry and Behavioral Neuroscience, University of Cincinnati College of Medicine, Cincinnati, OH USA; 7 Department of Psychiatry, Centre for Addiction and Mental Health, Institute of Medical Science, University of Toronto, ON Toronto, Canada; 8 Faculty of Computer Science, Dalhousie University, Halifax, NS Canada

**Keywords:** Bulimia nervosa, Lisdexamfetamine dimesylate, Qualitative research, Stimulant, Pharmacotherapy

## Abstract

**Background:**

There is emerging evidence that stimulants warrant further investigation as a treatment for bulimia nervosa (BN) including a recent open-label feasibility trial examining the use of lisdexamfetamine dimestylate (LDX) for BN. The current report presents the secondary outcomes and qualitative interview results from that feasibility trial. These outcomes explore several purported mechanisms that may explain how stimulants affect symptoms of BN: appetite, impulsivity, obsessive and compulsive symptoms, eating disorder psychopathology/impairment and reward-based decision-making.

**Methods:**

Twenty-three participants with BN received LDX for eight weeks. Questionnaires assessing appetite, impulsivity, obsessive and compulsive symptoms, eating disorder psychopathology and impairment were administered at baseline and post-treatment. Participants also completed a two-step reinforcement learning task to assess their decision-making. Semi-structured interviews took place at baseline, week 5, and follow-up.

**Results:**

Reductions in hunger, food-related impulsivity, obsessive and compulsive features, eating disorder psychopathology and impairment were found. However, reward learning, as far as it is assessed by the task, did not seem to contribute to the effect of LDX on BN symptoms. Qualitative analysis suggested four themes: (1) reprieve from the eating disorder, (2) improvement in function and quality of life, (3) renewed hope for recovery, and (4) ability to normalize eating.

**Conclusions:**

This report suggests several potential mechanisms by which LDX may reduce symptoms of binging and purging in those with BN. Importantly, due to the open-label design, we are unable to attribute findings to the medication. Instead, our results should be interpreted as hypothesis generating to inform future studies such as adequately powered randomized controlled trials.

*Trial registration* NCT03397446.

**Supplementary Information:**

The online version contains supplementary material available at 10.1186/s40337-023-00796-x.

## Abstract (plain language summary)

Recent research suggests that stimulant medications could be a potential treatment for bulimia nervosa (BN). Participants in this study took lisdexamfetamine dimesylate (LDX) for 8 weeks while their eating disorder symptoms and medical status were carefully monitored. As part of this study, twenty-three participants with BN completed several interviews, questionnaires and computer tasks at the start and end of treatment which were delivered to help researchers learn more about the how LDX impacts people with BN. Scores on questionnaires measuring different aspects of the eating disorder improved over time. Participants’ performance on the computer task which measures a type of decision making did not change during treatment. Interviews exploring participants’ experience taking LDX found four common themes: reprieve from the eating disorder, improvement in function and quality of life, renewed hope for recovery, and ability to normalize eating. This report suggests several potential ways LDX may reduce symptoms of binging and purging in those with BN. Importantly, due to the size and type of study, we cannot conclude that changes observed were a direct result of the medication. Instead, our results should be used to form new questions that can be explored by larger studies with controlled designs.

**Supplementary Information:**

The online version contains supplementary material available at 10.1186/s40337-023-00796-x.

## Background

The limited effectiveness of existing treatments [1] for bulimia nervosa (BN) warrants exploring novel interventions such as stimulants (see Keshen et al. [2] for a review). Robust evidence supports lisdexamfetamine dimestylate (LDX) as an effective treatment for binge eating in the context of binge eating disorder (BED) [3]. To date, no substantial clinical trials have specifically examined stimulant medication use in BN; however, case reports [2] have been promising and a recently published 8-week open-label feasibility trial found LDX was associated with a reduction in objective binge eating episodes and compensatory behaviors [4].

Stimulants are purported to impact the symptoms of binge eating in BED and BN through multiple mechanisms including reducing appetite, impulsivity, obsessive and compulsive symptoms, and affecting reward-based decision-making [3, 5–7].

Regarding reduced appetite, evidence from pre-clinical rat studies [8, 9] and LDX clinical trials for BED [3] suggest that stimulants have a general appetite suppressant effect. In the LDX for BED trials, up to a quarter of participants reported reduced appetite as an adverse event [6]. However, studies have not yet elucidated the degree to which appetite suppression moderates or mediates a reduction in binge eating symptoms in those treated with stimulants.

Animal models of impulsivity (e.g., delayed discounting task) have shown the tendency of binge-eating rats to act without regard for future consequences (non-planning impulsivity) compared to non-binge-eating rats [8, 10]. Moreover, the LDX for BED clinical trials by McElroy et al. [5] suggest that LDX improves impulsivity as measured by the Barratt Impulsiveness Scale (Version 11; BIS-11) [11]. Specifically, LDX was associated with motor impulsivity (tendency to act without thinking) and non-planning impulsivity improvements, though only at the 70 mg dose. More recently, Griffiths et al. [12] found that only the non-planning impulsivity subscale of the BIS-11 was associated with a decrease in binge episodes in those receiving LDX, suggesting that LDX may moderate reductions in binge frequency via concurrent reductions in that subtype of impulsivity only.

Binge eating has been associated with compulsive responding, or the tendency to engage in repetitive, or habitual behaviors despite negative consequences [6]. Reductions in obsessive and compulsive symptoms related to binge eating, as measured by the Yale-Brown obsessive-compulsive scale modified for binge eating (YBCOS-BE), have been reported with LDX [5].

Lastly, there has been research interest in reward-based decision-making deficits in BN; specifically, three putative mechanisms warrant further examination: (1) goal directed (model-based) vs. habitual (model-free) control, (2) learning rate, and (3) the exploration/exploitation trade-off (see Auer et al. [13] for a detailed description). ​​Since dopamine (DA) depletion is associated with these decision-making impairments [14–16], and stimulants increase intracellular DA levels, it is possible that stimulant mediated improvements in these cognitive deficits could explain BN symptom improvements in those treated with stimulants.

The current report presents the secondary outcomes and qualitative findings from the recently published open-label, 8-week LDX feasibility trial for adults with BN [4]. The primary efficacy outcome reported by Keshen et al. [4] was change in objective binge episodes and compensatory behaviors in the previous 28 days from baseline to post/end-of-treatment. In the intent-to-treat sample, there were reductions in objective binge episodes and compensatory behaviors from baseline to post/end-of-treatment (mean difference = − 29.83 binge eating episodes in the past 28 days, 95% confidence interval: − 3.38 to − 16.27; and mean difference = − 33.78 compensatory behavior episodes in the past 28 days, 95% confidence interval: − 48.74 to − 18.82, respectively). The aims of the current study were to examine changes in secondary measures of appetite, impulsivity, obsessive and compulsive symptoms, eating disorder psychopathology, functional impairment, and reward-based decision-making deficits (using a reinforcement learning task) during 8 weeks of LDX treatment. Additionally, the present study sought to explore participants’ subjective experiences with LDX during participation in the study from thematic analysis of semi-structured interviews. Hypotheses should be reserved for randomized control trials and not feasibility studies [17]; therefore, no specific hypotheses were made.

## Methods

### Study design

The study is an open-label, 8-weeks feasibility study utilizing a dose-optimization design [4] with both quantitative and qualitative data collection methods. The research conformed to the International Conference of Harmonization and Good Clinical Practice guidelines and was approved by Health Canada and the Nova Scotia Health Research Ethics Board. All participants provided written informed consent.

### Participants

Twenty-three participants with moderate to extreme BN were enrolled (female = 23; white = 95.65%; mean age = 26.83, *SD* = 7.96), with 18 completing the study (*n* = 2 withdrawn by investigator; *n* = 3 dropout). Please note that while one participant prematurely dropped out, they did so for non-exclusionary reasons, took their maintenance dose of LDX for 39 days, and completed their final data collection. As such, they are included in the completer analysis. See Dixon [18] for additional details on sample characteristics. Detailed information about participant enrollment and inclusion/exclusion criteria can be found in Keshen et al. [4].

### Quantitative outcome measures

The measures for assessing quantitative outcomes included:

The Three Factor Eating Questionnaire (TFEQ) [19] which is a 51-item self-report questionnaire measuring three dimensions of eating behavior (cognitive restraint, disinhibition, and hunger). Item response formats include true/false or Likert style responses (0–4 or 0–5). Each dimension has satisfactory internal consistency (α = 0.85–.93). The Hunger subscale of the TFEQ is a measure of the perceived feeling of hunger and its behavioural consequences. The Restraint subscale of the TFEQ evaluates dietary restraint and conscious mechanisms for restraining food intake.

The Barratt Impulsiveness Scale, Version 11 (BIS-11) [11] is a 30-item self-report measure of trait impulsivity. Items are scored from one (rarely/never) to four (almost always/always), resulting in a global score and subscales for attentional, motor, and non-planning impulsivity. Internal consistency has been shown to be acceptable.

The Yale-Brown Obsessive-Compulsive Scale Modified for Binge Eating (YBOCS-BE) [20] is a modified version of the Yale-Brown Obsessive-Compulsive Scale which assesses urges, thoughts, impulses, and compulsions related to binge eating. The YBOCS-BE is an interview measure and composed of 10 items scored from 0 (no symptoms) to 4 (extreme symptoms). With permission from the scale developer, the YBOCS-BE was further modified for BN (i.e., ‘purge’ was inserted following ‘binge’ throughout the measure).

The Eating Disorder Examination 17.0D (EDE) [21] was selected to measure eating disorder psychopathology. The EDE assesses psychopathology on four subscales (Restraint, Eating Concern, Shape Concern and Weight Concern) and provides a global score [21]. A systematic review by Berg et al. [22] reported internal consistency coefficients of EDE subscales in clinical eating disorder (ED) populations ranging from 0.64 to 0.85.

The Clinical Impairment Assessment questionnaire (CIA) [23] is a 16-item self-report measure of the severity of psychosocial impairment due to eating disorder features. Items cover impairment in domains of life that are typically affected by eating disorder psychopathology. A cut off score of 16 has shown to be an appropriate predictor of ED case status [23]. The CIA has excellent internal consistency, Cronbach’s alpha has been reported as 0.97 [23].

The Two-Step Reinforcement Learning Task [24] is a computer-based task that prompts users to make a series of choices between two stimuli. Each choice deterministically transitions to a second-stage state that is associated with a fluctuating reward payoff. Task-related decisions were used to assess participants’ relative degree of decisional goal-directed (model-based) versus habitual (model-free) behavioural control, learning rate and exploration/exploitation. A detailed description of the reinforcement learning task is presented in the Additional file 1.

### Qualitative interviews

Semi-structured interviews were completed at baseline, following medication titration (week 5), and during the follow-up visit. Baseline interviews focused on expectations for treatment with LDX and prior experiences with ED treatments. The week 5 and follow-up interviews explored participants’ experiences with the medication for symptoms related to their BN. The interview guide was developed by LD and refined by ARK and other team members (see Additional File 1 for full interview guides). Participants who did not complete the study were contacted for an interview at the time of discontinuation. All interviews were audio-recorded, transcribed verbatim by LD, and checked for accuracy by LD and SIA.

### Procedure

Participants were recruited through online advertisements, local classifieds, study posters, and through an ED clinic. Following informed consent, participants had a medical and psychiatric assessment to determine eligibility. The 8-week trial began with a flexible 4-week titration period to 50 mg or 70 mg of LDX, followed by a 4-week maintenance period [4]. The reinforcement learning task was administered at week 1 (baseline), week 2, week 9 (post-treatment) and week 10 (follow-up). All other quantitative measures were administered at baseline and week 9 (post-treatment). The dosage protocol and additional procedural details about screening, treatment, and follow-up phases are found in Keshen et al. [4].

### Statistical and qualitative analysis

Feasibility studies should not conduct null hypothesis significance testing without adequate power [17]. Given our relatively small sample size and feasibility study design, effect sizes were calculated using Cohen’s *d*, with 0.20, 0.50, and 0.80 corresponding to small, medium, and large effect sizes, respectively. Intent-to-treat (ITT) results compared post or last observation carried forward (LOCF) to baseline, while Completer Sample (CS) results compared post to baseline. For the reinforcement learning outcomes, computational learning models were fit to subjects’ trial-by-trial behaviours to quantify (A) the likely decision-making strategy being used and (B) the degree to which specific model parameters, such as model-based/model-free control, exploration-exploitation balance, and learning rate were used at each time point (see Additional File 1 for details). This was performed by constructing reinforcement learning models that mirrored the participants’ trial-by-trial behavioral data.

Qualitative data analysis followed Braun and Clark’s [25] approach to thematic analysis. Individual transcripts were inductively coded by LD through an iterative process of coding and revising codes based on data from additional transcripts. Codes were regularly reviewed and discussed amongst team members. Themes were developed through grouping codes and examining patterned meaning in the data.

## Results

Quantitative findings including means, standard deviations and Cohen’s *d* for all outcome measures are presented in Table 1. From sections “Hunger and cognitive restraint” to “Eating disorder psychopathology and impairment” quantitative and qualitative findings are integrated in a joint analysis format to describe the following outcomes: (A) hunger^1^ and cognitive restraint, (B) impulsivity, (C) obsessive and compulsive features and (D) eating disorder psychopathology and impairment. The “Reward-based decision making” section describes the results related to three putative reward-based decision-making deficits in BN (goal directed vs. habitual control, learning rate, and the exploration/exploitation trade-off). Finally, the “Thematic analysis” section summarizes the thematic analysis from qualitative interview data.

**Table 1:** Change in outcomes for ITT and completer samples during 8-weeks of lisdexamfetamine dimesylate treatment

Measure	Intent-to-treat sample (n = 23)					Study completers (n = 19)				
	Baseline		Post or LOCF		Cohen’s *d*	Baseline		Post		Cohen’s *d*
	*M*	*SD*	*M*	*SD*		*M*	*SD*	*M*	*SD*	
Hunger and cognitive restraint										
TFEQ restraint	12.52	5.26	10.57	4.99	0.38	12.47	5.33	9.68	4.57	0.56
TFEQ hunger	8.22	3.81	3.96	3.69	1.14	8.00	3.87	3.42	3.72	1.21
Impulsivity										
TFEQ disinhibition	12.74	2.49	6.26	4.27	1.85	12.74	2.57	5.16	3.67	2.31
BIS-11 total	65.26	8.80	63.87	11.61	0.13	65.11	7.89	62.84	11.12	0.24
BIS-11 attention	17.83	4.42	17.48	4.61	0.08	17.58	4.44	17.12	4.40	0.10
BIS-11 motor	23.04	3.87	22.52	4.09	0.13	23.00	3.51	22.16	4.03	0.22
BIS-11 NP	24.39	3.86	23.87	4.66	0.12	24.53	3.64	23.58	4.39	0.24
Obsessive compulsive features										
YBOCS-BE total	22.30	3.42	5.83	4.33	4.22	22.63	2.99	4.89	3.60	5.36
YBOCS-BE (O)	10.97	1.62	3.52	2.78	3.27	11.00	1.41	2.68	2.06	4.71
YBOCS-BE (C)	11.39	2.04	2.65	2.23	4.09	11.63	1.86	2.21	1.99	4.89
Eating disorder psychopathology and impairment										
EDE restraint	1.76	1.41	0.97	1.27	0.59	1.53	1.38	0.58	0.91	0.81
EDE eating concern	2.23	1.28	1.03	1.26	0.94	2.01	1.25	0.57	0.68	1.43
EDE shape concern	3.48	1.66	2.06	1.67	0.85	3.19	1.67	1.47	1.09	1.22
EDE weight concern	2.79	1.56	1.63	1.40	0.78	2.57	1.61	1.16	0.99	1.06
EDE global score	2.56	1.29	1.42	1.27	0.89	2.32	1.26	0.94	0.69	1.36
CIA	32.42	8.04	13.04	12.17	1.88	31.72	7.76	9.00	8.15	2.86

Cohen’s *d* reflects differences between baseline and post or LOCF measurements

*TFEQ *  Three factor eating questionnaire, *YBOCS-BE* Yale Brown obsessive compulsive scale (modified for binge eating),* O* obsessions, *C*  compulsions), *BIS-11* Barratt impulsiveness scale, *NP* non-planning, *EDE* eating disorder examination, *CIA* clinical impairment assessment

### Hunger and cognitive restraint

Both ITT and CS demonstrated large score reductions on the TFEQ Hunger subscale and small-medium reductions in the TFEQ Restraint subscale. In qualitative interviews, participants described overall improvement in the regulation of hunger and ability to implement more flexible and structured meals (see Theme 4 and associated quotes in Table 2 for examples).

**Table 2:** Description of four themes and example quotes

Theme	Participant quotes
**Theme 1: Reprieve from the Eating Disorder** Participants reported experiencing a reprieve from ED behaviors, urges, and cognitions while taking LDX. Participants describe being surprised by how easy it now felt to abstain from binging and purging. They reported that stress from their ED and from the cycle of binge eating and purging was alleviated.	**Quote #1:** “It’s a weird adjustment to make, suddenly to not just have so much of my day consumed by that, that cycle, right? Like every day too…, I was never feeling good, I was just taken over by it and I didn’t have like a desire to do anything really or like put myself in situations where I’d have to do things, and to not have that kind of hanging over me anymore was a strange feeling but a good one. It was like suddenly having like this huge weight not there anymore.” [Participant #4] **Quote #2:** “Yeah it’s been interesting to see how life is without having to worry about binging every day and purging every day and just obsessive thoughts about being hungry all the time and then being upset because you don’t want to eat bad food but you want to. So yeah, ‘cause I experienced that for like 10 years straight so it’s been like a lot of stress lifted from not having those thoughts.” [Participant #13]
**Theme 2: Improvement in Function and Quality of Life** Participants reported improvements in many domains affected by their ED. For example, they reported improved ability to function at work and school and engage with others socially. They described experiencing increased feelings of connection with their families and loved ones, and improvements to their mood.	**Quote #3:** “I didn’t even, even answering the questions now compared to the beginning… I didn’t really know how much it [the eating disorder] influenced my life until it wasn’t as prevalent and it wasn’t there every single day. Like that drive and that obsessive thought process behind it, I didn’t even realize that was a component to it so just realizing that it can be different than it was [has been the best part about participating].” [Participant #14] **Quote #4:** “Just like a) financially definitely is a big one. While I was on [the medication] at least, being able to go out with my friends, and you could go out to eat or whatever and you wouldn’t have any issues, that is a big thing. So like socially too, and with my family things were a lot better too… I think probably because I didn’t realize before how you do become agitated when you are always having these thoughts and you’re not agitated at the people, you’re just agitated in general. So I think I didn’t realize how bad that was [before] versus now.” [Participant #1] **Quote #5:** “Oh my goodness, so radically really. Like I just, I just feel so much more in control of who I am and I feel like I’m a better mom, I feel like I’m better at my job, I, you know, I just feel, I feel better, I feel, you know. I don’t know how else to put it but it’s, it’s really changed.” [Participant #15]
**Theme 3: Renewed Hope for Recovery** Participants often reported that prior to the trial, they felt hopeless and had little confidence in their ability to stop binge eating and purging or to fully recover from their ED. Participants expressed that taking the medication and experiencing the subsequent improvements in ED behaviors during the trial offered a newfound sense that recovery was possible.	**Quote #6:** “I felt like I had my life back. Like, and that makes me get super emotional saying that, it sounds so corny, but it’s like I haven’t been able to do what I’ve been doing the last two months in years, and I can remember how driven and how ambitious and how hard working I used to be, and that all got taken away with the eating disorder…, and I hadn’t realized how much the eating disorder itself had taken away from my life, and like being on this medication just made everything easy. It made my like life function, I could do what I wanted to do, I didn’t have to even think about wanting to binge and purge, which I haven’t had in years…, I could see the light and the hope of what life could then be like again …, I don’t know if I’ve ever really gotten that since this eating disorder started.” [Participant #3] **Quote #7:** “I’m feeling a little bit more hopeful that I can change this, there was a time that I felt, you know, like this will just be how I, how life is, which was a very sad thought to think but I’m starting to get some, see the light at the end of the tunnel and see that you know, change is possible and um I can have a life free of this hopefully.” [Participant #16] **Quote #8:** “It’s given me a lot of hope and that’s been a really exciting thing. I felt really lost for a really long time and it’s given me, and in some aspects more than others definitely, but it’s given me a sense of normalcy that I really enjoy.” [Participant #17]
**Theme 4: Ability to Normalize Eating** Participants described an increased ability to implement more consistent meal structure and/or to improve existing meal structure while on the medication. Participants reported increased flexibility and freedom around food that allowed them to eat a greater variety of food, as well as increase consumption of “fear foods”. In some instances, participants reported that the medication normalized their appetite, either increasing their ability to experience hunger and fullness cues or normalizing their “insatiable” appetites.	**Quote #9:** “I feel like the medication was like a nice reset so I can like, I got myself on this whole plan of I’ll eat every 3 hours and I’ll eat vegetables or whatever and I was just able to like start fresh you know? And now I can hopefully continue that for the rest of my life.” [Participant #2] **Quote #10:** “Prior to the study, say if I was trying to stop binging and purging I think I was super restrictive on what I would eat when I didn’t really realize that back then. There was foods I almost had deemed bad foods and now, slowly over the last couple of weeks I introduce different things that maybe prior to this I wouldn’t have eaten if I was trying to not binge and purge.” [Participant #1] **Quote #11:** “I think [my appetite] probably has gone down a little bit but I’ve also been able to sort of listen to my hunger cues in ways that I wasn’t able to before. Even though I still sort of eat no matter what, but I’m aware of them. Sometimes I would try to listen to my hunger cues before but they were so out of whack I had no idea. I didn’t even really know what it felt like to be hungry or full or yeah, so it’s definitely sort of helped me get to a stable place where I can start sort of remembering what that feels like.” [Participant #18].

### Impulsivity

In the ITT sample, total and subscale scores of the BIS-11 remained consistent. In the CS, small reductions were observed for the total score and motor and non-motor planning subscales of the BIS-11. Negligible changes were observed for the BIS-11 attention subscale. Both ITT and CS demonstrated large reductions on the TFEQ disinhibition subscale (eating-related impulsivity). In qualitative interviews, participants described a greater degree of control over their binge eating and purging behaviors while taking LDX, including increased ability to consider the outcomes of binge eating/purging instead of acting impulsively on these urges (see Quote #1 in Table 2 for an example).

### Obsessive compulsive features

Both ITT and CS demonstrated large reductions in total scores and subscale scores of the YBOCS-BE. In qualitative interviews, participants reported similar reductions or complete absence of bulimia-related obsessive and compulsive features (see Quote #2 in Table 2 for an example).

### Eating disorder psychopathology and impairment

In the CS and ITT sample, reductions in EDE scores corresponding to large effect sizes were observed on all subscales and the Global score of the EDE apart from the Restraint subscale for the ITT sample where a medium effect size was found. During qualitative interviews, participants often described a reduction or absence of eating disorder thoughts (e.g., thoughts about food, eating, urges to binge/purge, shape and weight). There were mixed responses regarding thoughts about body image/shape/weight as some participants continued to experience these thoughts while others noted these thoughts were less prominent or impactful.

Both the CS and ITT sample demonstrated reduced impairment corresponding to large effect sizes. See Table 2, Theme 2 and associated quotes for details on changes to impairment and quality of life reported by participants.

### Reward-based decision making

At all time points, participants used habitual (model-free) control during the completion of the two-step task (see Additional File 1). This meant that participants were more likely to select previously rewarded actions instead of acknowledging the underlying, causal structure of the task. Participant learning rate was negligible across all time points (i.e. participants were unable to adjust their prediction of task outcomes over time; see Additional File 1). As LDX did not affect learning rate, it is irrelevant to report on participants’ exploration/exploitation tradeoff. This is because the participants were found to be insensitive to reward contingencies. As a result of this, they would not be able to differentiate between selecting the option of highest expected value (exploitation), and exploration of the environment for potentially greater rewards.

### Thematic analysis

Findings from the thematic analysis include four main themes: (1) reprieve from the ED, (2) improvement in function and quality of life, (3) renewed hope for recovery, and (4) ability to normalize eating. See Table 2 for a description of these themes and sample participant quotes.

##  Discussion

In this open-label feasibility study with 23 participants, reductions in hunger (without a corresponding increase in restraint as per the EDE and TFEQ), eating-related impulsivity, bulimia-related obsessions and compulsions, eating disorder psychopathology, and impairment were observed. The expected improvements in reward-based decision-making were not observed.

###  Hunger and restraint

Based on the TFEQ hunger subscale score changes and Themes 1 and 4, participants experienced a reduction in hunger that was often described as ‘excessive’ at baseline. In other words, these participants interpreted their hunger as *too high* at baseline and construed the LDX as reducing their hunger to normalized levels. However, since hunger measurement in this study was subjective, it is not clear whether participants were objectively experiencing excessive hunger at baseline. Instead, they may have been experiencing subjective distress about normal degrees of hunger and the LDX may have suppressed their hunger to below normal levels.

Notably, despite observing decreased hunger per the TFEQ and decreased appetite (as described by Keshen et al. [4]), levels of restraint measured by the EDE and TFEQ decreased during the study. This is consistent with results discussed in Theme 4: Ability to Normalize Eating, where participants reported having more flexibility and freedom around food, allowing for less restrained eating. In the interviews, participants often reported feeling more purposeful about their eating choices alongside reduced urges to binge and binge eating behaviours. As a result, participants may have been less driven to restrict food intake to compensate for loss of control eating (i.e., binging). This finding is reassuring because dietary restriction and cognitive restraint are known to worsen or maintain BN [26].

### Impulsivity

Results demonstrated a large reduction in TFEQ Disinhibition (food/eating specific impulsivity), but only a small or negligible reduction in BIS-11 impulsivity scales (general trait impulsivity). This difference may be accounted for by the degree to which each type of impulsivity was present in the sample at baseline. The average TFEQ Disinhibition score was clinically elevated at baseline and thus had the potential for significant improvement, while the BIS-11 impulsivity scales were not elevated at baseline relative to clinical norms, thereby leaving less potential for such improvement. Notably, participants with ADHD were excluded from the trial (i.e., high trait impulsivity individuals).

Our results were not consistent with other studies that found improvements in non-planning impulsivity [27] and non-planning/motor [5] subscales of the BIS-11 in BED participants treated with LDX. One explanation for this difference is that other studies [5, 27] may have had higher levels of baseline trait impulsivity in their samples and limited exclusion of those with ADHD. Alternatively, this difference could represent a discrepancy in the significance of trait-impulsivity as a moderator/mediator of LDX effect in those with BN versus BED.

### Obsessive compulsive features

The YBOCS-BE and qualitative interviews elucidated decreased presence and impact of both obsessions and compulsions related to binging and purging. These findings were consistent with decreases in binge-related obsessions and compulsions in the BED LDX trials [5]. Mechanistically, it is possible that LDX-induced appetite reduction lessens the drive to eat, thereby diminishing obsessive thoughts and compulsive urges to engage in binge eating and purging. It is also possible that with fewer binge eating and compensatory behaviours, participants had more consistent nourishment, resulting in fewer hunger-driven obsessions and compulsions to binge eat [28]. Plausibly, LDX may have also mediated *non-appetite* related changes to brain regions involved in compulsivity (e.g., the dorsal striatum [29]).

### Eating disorder psychopathology and impairment

Reductions on all subscales and the Global score of the EDE were observed and these quantitative results are supported by qualitative findings. Participants generally described reduced frequency and intensity of eating disorder cognitions during the study. For some, one exception was concerns about weight and shape. Although reductions in scores on the Weight Concern and Shape Concern subscales were observed on quantitative measures overall, participants described varying experiences with changes in shape/weight concerns. Responses from participants varied from reporting no change to the degree of concern about their weight/shape and body image to reporting thoughts and concerns were absent or had decreased in frequency and importance. Given that overevaluation of weight and shape is known to maintain EDs [30] and contribute to relapse [31], it is important that future studies examining the use of LDX for BN continue to assess this variable during treatment. Should future research support the use of LDX for BN, combining LDX with psychotherapy which directly targets shape/weight concern may be beneficial for addressing ongoing overevaluation of shape and weight.

Reduced CIA scores corresponding to large effect sizes were observed from Baseline to Post in both the CS and ITT sample. This finding was also captured in qualitative interviews (e.g., Theme 2: Improvements in Function and Quality of Life); for example, participants often reported improvements to various functional domains (e.g., school, work, socially). Since the CIA focuses on impairment from the eating disorder it is possible that the reprieve from the eating disorder participants experienced (i.e., Theme 1) could account for reduced impairment. Having freedom from the consuming nature of eating disorder thoughts, urges, and behaviours may have allowed participants to engage in valued activities. It is also possible that the described functional improvements may be explained by stimulant mediated effects unrelated to changes in ED symptoms (e.g., improved mood).

### Reward-based decision making

Unexpectedly, reward-based decision making (as measured by a reinforcement learning task) did not improve with LDX treatment and was not associated with decreasing binge/purge behaviours. One explanation is that LDX does not mediate/moderate BN symptom reduction through reward-based decision-making processes. Alternatively, while it is unlikely that the reinforcement task lacked ecological validity, this should also be acknowledged as a possible reason why participant reward learning did not relate to a reduction in BN symptoms.

### Thematic analysis

Participants experienced an increased ability to normalize their eating (i.e., Theme 4) and hope for recovery (i.e., Theme 3), both of which have been found to predict ED recovery [28, 32]. Moreover, results from Theme 2 suggest that changes in symptomatology translated to changes in their ability to function in social, work, and academic domains. Participants also reported improvements to their quality of life. These findings further support the rationale for conducting adequately powered RCTs that explore the use of LDX for BN.

### Limitations

Our study had a small sample size, was open-label, and not placebo-controlled. Additionally, the duration of treatment (8 weeks) and follow-up (1 week) in the trial was relatively short. Further, stringent inclusion/exclusion criteria limited the sample to a narrow subset of individuals with BN. For example, inclusion was limited to only those with moderate to extreme severity of illness, a BMI between 21 and 30 kg/m^2^, limited psychiatric comorbidities, no history of anorexia nervosa, and minimally restrictive eating patterns at baseline. Full inclusion/exclusion criteria are listed in the Keshen et al. [4] supplemental file.

The results presented should be interpreted cautiously due to the absence of a control group and it should be recognized that participants were carefully monitored during treatment and aware of criteria for treatment discontinuation such as weight loss or increased dietary restriction based on weekly/bi-weekly assessment. Considering these limitations, the results should be interpreted as preliminary, and not as sufficient evidence to recommend routine clinical use of LDX for BN.

###  Conclusion

Quantitative and qualitative results from this feasibility study suggest that participants experienced reduced hunger (without a corresponding increase in restraint), eating-related impulsivity, bulimia-related obsessions/compulsions, eating disorder psychopathology, and impairment while participating in the trial. Moreover, participants described experiencing a reprieve from their ED, improvement to their functioning and quality of life, and renewed hope for recovery from BN. Ideally, these findings will help generate hypotheses for future studies that explore mechanistic pathways and clinical outcomes with stimulants, such as LDX, in the management of this complex and debilitating disorder.

### Supplementary Information

**Additional file 1.** Information about the reinforcement learning task and complete qualitative interview guides.

## Data Availability

The datasets used and/or analyzed during the current study are available from the corresponding author on reasonable request.

## Abbreviations

BED: Binge eating disorder
BIS-11: Barratt Implulsiveness Scale Version 11
BN: Bulimia nervosa
CIA: Clinicial impairment assessment
CS: Completer sample
DA: Dopamine
ED: Eating disorder
EDE: Eating Disorder Examination 17.0D
ITT: Intent-to-treat
LDX: Lisdexamfetamine dimesylate
LOCF: Last observation carried forward
RCT: Randomized control trials
TFEQ: Three Factor Eating Questionnaire
YBOCS-BE: Yale-Brown obsessive-compulsive scale modified for binge eating

## Footnotes

1 Hereafter, the concepts of hunger and cognitive restraint will reflect, or are proxy measures for, the concept of ‘appetite’.
