# Correction: Secondary outcomes and qualitative findings of an open-label feasibility trial of lisdexamfetamine dimesylate for adults with bulimia nervosa

**DOI:** 10.1186/s40337-023-00926-5

**Published:** 2023-11-16

**Authors:** Laura Dixon, Sara Bartel, Victoria Brown, Sarrah I. Ali, Susan Gamberg, Andrea Murphy, Katherine L. Brewer, Susan L. McElroy, Allan Kaplan, Abraham Nunes, Aaron R. Keshen

**Affiliations:** 1 Eating Disorder Program, Nova Scotia Health Authority, Halifax, NS Canada; 2 Department of Psychiatry, Dalhousie University, Halifax, NS Canada; 3 College of Pharmacy, Dalhousie University, Halifax, NS Canada; 4 College of Doctoral Studies, University of Phoenix, Tempe, AZ USA; 5 Lindner Center of HOPE, Mason, OH USA; 6 Department of Psychiatry and Behavioral Neuroscience, University of Cincinnati College of Medicine, Cincinnati, OH USA; 7 Department of Psychiatry, Centre for Addiction and Mental Health, Institute of Medical Science, University of Toronto, Toronto, ON Canada; 8 Faculty of Computer Science, Dalhousie University, Halifax, NS Canada

**Correction:Journal of Eating Disorders (2023) 11:81** **https://doi.org/10.1186/s40337-023-00796-x** 

The original article [1] has incomplete funding information. The sponsorship from Takeda Pharmaceutical Australia to cover the Article Processing Charge was not mentioned in the Funding note. The correct Funding note is as follows:

**Funding**

This work was supported by the Nova Scotia Health Authority Research Fund and the Dalhousie Department of Psychiatry (Award No. 1023016). The Nova Scotia Health Authority Research Fund and the Dalhousie Department of Psychiatry were not involved in the design of the study, data collection, analysis and interpretation, or in writing the manuscript. The article has received sponsorship from Takeda Pharmaceutical Australia to cover the Article Processing Charge.

The original article [1] has been corrected.
