# Supplementary material for: Secondary outcomes and qualitative findings of an open-label feasibility trial of lisdexamfetamine dimesylate for adults with bulimia nervosa
Source: J Eat Disord. 2023 May 22;11:81. doi: 10.1186/s40337-023-00796-x (PMC10204259; doi:10.1186/s40337-023-00796-x)
Supplement: Supplementary file 1 — Additional file 1. Information about the reinforcement learning task and complete qualitative interview guides. [file 40337_2023_796_MOESM1_ESM.pdf]

Supplemental Files

Contextual Bandit Reinforcement Task

Block (and their code names)	Description
ID Collection	Participant's study ID and Date are input and held as a global variable to which other data from the task are linked.
"Real Trials"	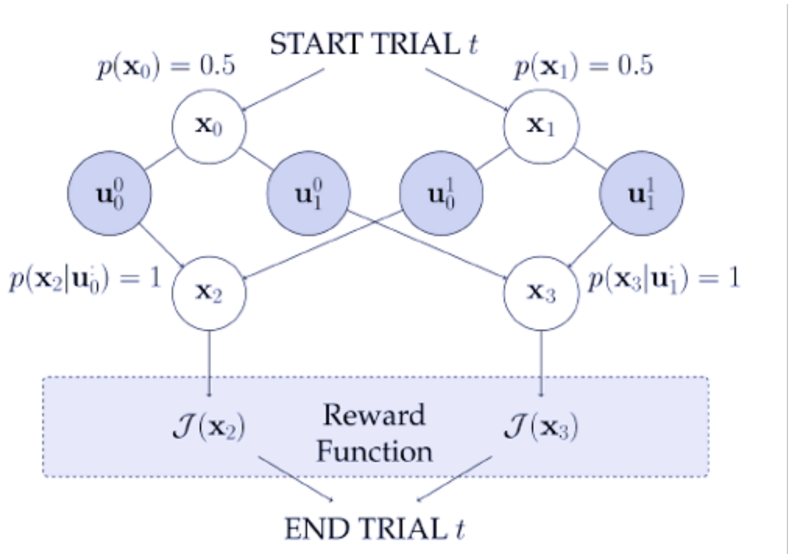<br>Individuals start trial t by entering either state x_0 (state with spaceships A and B) or state x_1 (state with spaceships C and D). If participant starts in state x_0, they may choose either action u_0^0 (spaceship A) or u_1^0 (spaceship B). If participant starts in state x_1, they can choose either action u_0^1 (spaceship C) or u_1^1 (spaceship D). At each trial, spaceship pairs are placed on either the left or right of the screen according to a probability of 0.5. The choice of spaceship leads deterministically to state x_2 (one of the planets) or state x_3(the other planet) where the participant receives a reward if he or she presses the "space bar" key on the keyboard. Between trials, participants are presented with a fixation cross in order to retain attention to the centre of the screen.

Reinforcement Learning Models

Model	Component	Equation	Parameters
Hybrid	Policy	Softmax $\varsigma(x) = \frac{exp\{\beta\ x_i\}}{\sum_j \quad exp\{\beta\ x_j\}}$	β:: Inverse softmax temperature (choice consistency)
	Value function	Model-free (Q^{MF}): SARSA<br>Model-based (Q^{MB}): Bellman<br><br>Integration:<br>$Q = \omega Q^{\mathrm{MB}} + (1 - \omega)Q^{\mathrm{MF}}$	Model free:<br>Learning rate: $0 \leq \alpha \leq 1$<br><br>Integration:<br>Model-based/model-free balance: $0 \leq \omega \leq 1$
Model-free	Hybrid model with ω fixed to 0		
Model-based	Hybrid model with ω fixed to 1		

Fitting the models to the participants' behavioural data using expectation maximization (doi: 10.1371/journal.pcbi.1002028) yielded an n_{subject} x n_{model} matrix of approximations to the logarithmic model evidence, which was then submitted to the Bayesian Model Selection procedure (Rigoux et al., 2014) in order to identify the most probable model that explained the aggregate group's behavioural data.

The models investigated were a model-free reinforcement learning algorithm developed using State-Action-Reward-State-Action, SARSA (λ) temporal difference learning (R. Sutton et al., 1999; R. S. Sutton & Barto, 1998), a model-based reinforcement learning algorithm using

Bellman equation (Bellman & Rand Corporation, 1957), and a hybrid model with model-based and model-free subcomponents. In the hybrid model, the relative weighting of participant model-based and model-free strategies was parameterized by w, where 1 indicates pure model-based learning, and 0 denotes pure model-free learning

The definitions of these functions are as follows:

● α: Learning rate: a coefficient which indicates how quickly an agent updates their state-action reward expectations. Participants with a high learning rate would be able to acquire reward related information about their actions quickly.

● β: Inverse softmax temperature parameter (an index of the amount of choice randomness). Typically, a higher inverse softmax temperature could reflect a more exploitatory choice strategy. When $\beta \rightarrow 0$, choice consistency decreases and actions become more random.

● ρ: Perseveration: The tendency to repeat a previously selected action regardless of the action's value. Participants with high perseverance, would tend to repeat choices made on the previous trial of the task.

Bayesian Model Selection Results

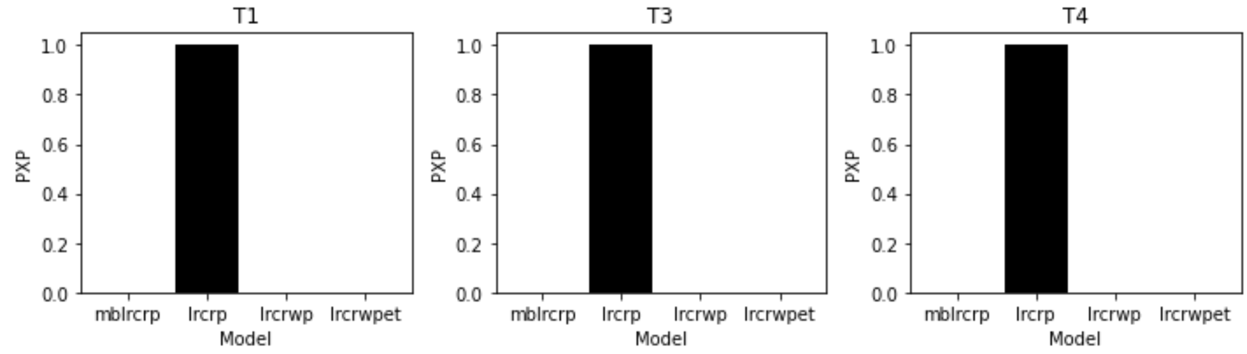

Note: T1= Baseline; T3= Maintenance dose; T4=Off study medication; mblrcrp= Model-based agent with learning rate, inverse softmax temperature, and perseveration; lrcrp= Model-free agent with learning rate, inverse softmax temperature, and perseveration; lrcrwp=Hybrid agent with learning rate, inverse softmax temperature, and perseveration; lrcrwpet=Hybrid agent with learning rate, inverse softmax temperature, perseveration, and eligibility trace; pxp=protected exceedance probability.

Estimation of Model Parameters (Model-Free Agent with Learning Rate, Inverse Softmax Temperature and Perseveration)

Participant ID	Time	α	β	ρ
A	T1	0.0018	45.06	-0.70552
A	T3	0.0140	0.0104	-0.1991
A	T4	0.0014	88.43	-0.2239
E	T1	0.0012	46.81	-0.3450
E	T3	0.0028	41.42	-0.4575
E	T4	0.0106	9.295	-0.3933
F	T1	0.0203	1.086	0.9299
F	T3	0.0010	105.0	-0.2246
F	T4	0.0017	81.10	-0.6789
G	T1	0.0029	50.29	-0.1660
G	T3	0.0026	36.79	-0.0627
G	T4	0.0016	89.94	-0.4221
H	T1	0.0021	68.50	-0.4953

H	T3	0.0051	11.014	-0.3683
H	T4	0.0027	80.06	-0.2336
I	T1	0.0049	30.68	-0.0593
I	T3	0.0078	48.95	-0.5278
I	T4	0.0043	56.43	-0.7028
J	T1	0.0337	0.5628	0.1008
J	T3	0.0145	8.044	-0.5059
J	T4	0.0008	99.30	-0.1692
M	T1	0.0013	57.02	-0.3713
M	T3	0.0415	0.6696	-0.0420
M	T4	0.0039	56.73	-0.3585
N	T1	0.0057	34.58	-0.2432
N	T3	0.0011	68.50	-0.5610
N	T4	0.0007	98.75	-0.3042
O	T1	0.0010	42.41	0.0984
O	T3	0.0059	42.29	-0.4663

O	T4	0.0003	115.6	-0.0308
P	T1	0.0021	45.49	-0.6029
P	T3	0.0019	70.71	-0.5259
P	T4	0.0016	92.27	-0.7287
Q	T1	0.0014	45.26	-0.3082
Q	T3	0.0035	16.28	-0.4172
Q	T4	0.0026	88.98	-0.7585
S	T1	0.0077	18.36	0.0014
S	T3	0.0056	22.86	-0.5736
S	T4	0.0019	86.56	-0.4512
T	T1	0.0009	59.42	-0.2453
T	T3	0.0019	83.09	-0.3211
T	T4	0.0059	55.21	-0.7120
U	T1	0.0016	60.28	-0.2433
U	T3	0.0041	11.61	-0.8500
U	T4	0.0008	93.56	-0.6122

V	T1	0.0015	57.70	-0.1091
V	T3	0.0014	77.039	-0.4418
V	T4	0.0008	92.83	-0.3187
W	T1	0.0007	50.94	-0.1423
W	T3	0.0013	125.5	-0.6085
W	T4	0.0212	1.336	-0.0419

Note. T1= Baseline; T3= Maintenance dose; T4=Off study medication; α = learning rate; β= inverse softmax temperature; ρ= perseveration.

References

Bellman, R., & Rand Corporation. (1957). *Dynamic Programming*. Princeton University Press.

Rigoux, L., Stephan, K. E., Friston, K. J., & Daunizeau, J. (2014). Bayesian model selection for group studies—Revisited. *NeuroImage*, *84*, 971–985. https://doi.org/10.1016/j.neuroimage.2013.08.065

Sutton, R., McAllester, D., Singh, S., & Mansour, Y. (1999). Policy gradient methods for reinforcement learning with function approximation. *Advances in Neural Information Processing System*, *12*, 1057–1063.

Sutton, R. S., & Barto, A. G. (1998). *Reinforcement learning: An introduction*. MIT Press.

Qualitative Interview Guides

Interview 1 – Expectations

START.

Thank you for meeting with me today. My name is Laura and I'm a research assistant working on this research study. I'm also a MSc student with Dalhousie's Department of Psychiatry. I will be conducting this interview today, as well as two additional interviews that will take place halfway through the study and at the end of the study. The purpose of these meetings is to gather information about your experience with the research study. As this is for research purposes, I will mainly be asking you questions, listening to your answers, and guiding the interview. I won't be able to provide too much feedback during the interview but if anything comes up that you'd like to discuss further with us, we can make a note about that and discuss it afterwards. The purpose of our meeting today is to understand your expectations as a participant in the research study. The interview should take approximately XX minutes. There will be an opportunity towards the end to ask questions and talk about anything not covered during the question period.

The audio of this conversation will be recorded for research purposes. The recording and the transcript will be transferred to a secure hospital drive where any identifying characteristics or mentions of your name will be deidentified. Only myself and the other members of the research team will have access to the recordings and transcripts.

I'm now going to start the recording.

You are free not to answer any questions you're not comfortable answering and you can choose to end the interview at any point.

Do you have any questions before we get started?

1. Can you tell me a little bit about why you want to participate in this study?

Probes: What are you hoping will change as a result of participating in the study?

Is there anything specific to your eating disorder symptoms that you hope will change?

Is there anything specific to your mood, emotions, or the way you feel that you hope will change?

Is there anything related to your day to day life or ability to function that you hope will change?

2. Have you ever received any type of treatment or support for your eating disorder (i.e., therapy, counseling, treatment at the hospital, a support group)? What type(s) of treatment or support have you received?

If treatment has been attended previously:

Probes: What was your experience like with ________ (treatment received)? Did you find the treatment was helpful/unhelpful? Can you give me some examples of what was helpful/unhelpful?

Do you think the study medication might help you? Do you have a sense of why you think/don't think the medication might help you?

Do you think the medication might help you more than the treatments you've tried in the past? Do you have a sense of why you think/don't think the medication would be more helpful/wouldn't be as helpful?

If treatment has never been attended previously:

Do you think the study medication might help you? Do you have a sense of why you think/don't think the medication might help you?

3. Is there anything else you would like to tell me about how you are feeling about participating in the study?

END

If there's nothing else you'd like to add, that brings us to the end of the questions for today. Thank you for taking the time to speak with me today, I appreciate your feedback. We'll now proceed to the next section of the study visit.

Stop recording.

Interview 2 – Midway

START.

Thank you for meeting with me today. As a reminder, my name is Laura and I'm a research assistant working on this research study. I'm also a MSc student with Dalhousie's Department of Psychiatry. The purpose of this meeting is to gather information about your experience with the research study so far. As this is for research purposes, I will mainly be asking you questions, listening to your answers, and guiding the interview. I won't be able to provide too much feedback during the interview, but if anything comes up that you'd like to discuss further with us, we can make a note about that and discuss it afterwards. The interview should take approximately XX minutes. There will be an opportunity towards the end to ask questions and talk about anything not covered during the question period.

The audio of this conversation will be recorded for research purposes. The recording and the transcript will be transferred to a secure hospital drive where any identifying characteristics or mentions of your name will be deidentified. Only myself and the other members of the research team will have access to the recordings and transcripts.

I'm now going to start the recording.

You are free not to answer any questions you're not comfortable answering and you can choose to end the interview at any point.

Do you have any questions before we get started?

1. How has your experience been participating in the research study so far?

If study medication not mentioned:

Probe: How has your experience been taking the study medication?

2. When you think about your day-to-day life before you started taking the medication and your life now, how would you say your life has changed?

Probes: Have any parts of your life gotten better? Worse?

What do you think the medication has helped you with the most?

What do you wish the medication was helping you with more?

If appetite or hunger is not discussed previously, or to clarify changes:

3. Since the beginning of the study, how would you describe any changes in your eating habits or behaviours?

Probes: Have you noticed any changes in your appetite or hunger specifically?

Have you noticed any changes in your binge eating and purging specifically?

Have you noticed any changes in your ability to think about the pros and cons of your behaviours before acting?

Overall, would you say the eating disorder symptoms have improved, stayed the same, or gotten worse since starting the medication?

Can you give me an example of what has _______ (improved, stayed the same, or gotten worse) since starting the medication?

If no changes identified: Can you tell me about how your eating habits or behaviours have stayed the same?

Probe: Is there anything about your eating habits or behaviours that you wish was changing?

4. Since the beginning of the study, how would you describe any changes in your mood, emotions, or the way you feel?

Probes: Overall, would you say your mood, emotions, or the way you feel has improved, stayed the same, or gotten worse since starting the medication?

Can you give me an example of what has _______ (improved, stayed the same, or gotten worse) since starting the medication?

If no changes identified: Can you tell me about how you think your mood, emotions, or the way you feel has stayed the same?

Probes: Is there anything related to your mood, emotions, or the way you feel that you wish was changing?

5. Since the beginning of the study, how would you describe any changes in your thoughts or the way you think about things?

If eating disorder thoughts are not mentioned: How would you describe any changes specific to your eating disorder thoughts?

If participant is unsure or asks for examples of eating disorder thoughts: Some examples would be thoughts about food or eating, thoughts about your weight or body, or urges to binge and purge.

Probe: Have you noticed any changes in the amount of time you spend thinking about food throughout the day?

If no changes are identified: Can you tell me about how you feel your thoughts or the way you think about things has stayed the same?

Probe: Is there anything related to your thoughts or the way you think about things that you wish was changing?

6. How are you feeling about continuing with the rest of the study?
7. Is there anything else you would like to tell me about how you have been feeling or doing since the beginning of the study?

END

If there's nothing else you'd like to add, that brings us to the end of the questions for today. Thank you for taking the time to speak with me today, I appreciate your feedback. We'll now proceed to the next section of the study visit.

Stop recording.

START.

Thank you for meeting with me today. As a reminder, my name is Laura and I'm a research assistant working on this research study. I'm also a MSc student with Dalhousie's Department of Psychiatry. The purpose of this meeting is to gather information about your experience with the research study. As this is for research purposes, I will mainly be asking you questions, listening to your answers, and guiding the interview. I won't be able to provide too much feedback during the interview but if anything comes up that you'd like to discuss further with us, we can make a note about that and discuss it afterwards. The interview should take approximately XX minutes. There will be an opportunity towards the end to ask questions and talk about anything not covered during the question period.

The audio of this conversation will be recorded for research purposes. The recording and the transcript will be transferred to a secure hospital drive where any identifying characteristics or mentions of your name will be deidentified. Only myself and the other members of the research team will have access to the recordings and transcripts.

I'm now going to start the recording.

You are free not to answer any questions you're not comfortable answering and you can choose to end the interview at any point.

Do you have any questions before we get started?

1. How has your experience been participating in the research study?

Probes: What would you say has been the best part about participating in the study?

What would you say has been the worst part about participating in the study?

If study medication is not mentioned:

Probse: How has your experience been taking the study medication?

2. When you think about your day-to-day life before you started taking the study medication and your life now - how would you say your life has changed?

Probes: Have any parts of your life gotten better? Worse?

What do you think the study medication has helped you with the most?

What do you wish the study medication helped you with more?

If appetite or hunger is not discussed previously, or to clarify changes:

3. Since the beginning of the study how would you describe any changes in your eating habits or behaviours?

Probes: Have you noticed any changes in your binge eating and purging specifically?

Have you noticed any changes in your ability to think about the pros and cons of your behaviour before acting?

Overall, would you say the eating disorder symptoms have improved, stayed the same, or gotten worse since starting the medication?

Can you give me an example of what has _______ (improved, stayed the same, or gotten worse) since starting the medication?

If no changes identified: Can you tell me about how your eating habits or behaviours have stayed the same?

Probe: Is there anything about your eating habits or behaviours that you wish was changing?

4. Since the beginning of the study, how would you describe any changes in your mood, emotions, or the way you feel?

Probes: Overall, would you say your mood, emotions, or the way you feel has improved, stayed the same, or gotten worse since starting the medication?

Can you give me an example of what has _______ (improved, stayed the same, or gotten worse) since starting the medication?

If no changes identified: Can you tell me about how you think your mood, emotions or the way you feel has stayed the same?

Probe: Is there anything related to your mood/emotions or the way you feel that you wish was changing?

5. Since the beginning of the study, how would you describe any changes in your thoughts or the way you think about things?

If participant is unsure or asks for examples of eating disorder thoughts: Some examples would be thoughts about food or eating, thoughts about your weight or body, or urges to binge and purge.

Probe: Have you noticed any changes in the amount of time you spend thinking about food throughout the day?

If no changes are identified: Can you tell me about the thoughts you are still having or how the way you think about things has stayed the same?

Probe: Is there anything related to the thoughts or the way you think about things that you wish was changing?

6. If you could, would you want to continue taking the medication? Why or why not?

7. Is there anything that would have made participating in the study easier for you?

8. Is there anything else you would like to tell me about how you have been feeling or doing since the beginning of the study?

END

If there's nothing else you'd like to add, that brings us to the end of the questions for today. Thank you for taking the time to speak with me. I appreciate your feedback. We'll now proceed to the next section of the study visit.

Stop recording.
